# Long-Lasting Insecticide Netting for Protecting Tree Stems from Attack by Ambrosia Beetles (Coleoptera: Curculionidae: Scolytinae)

**DOI:** 10.3390/insects11010008

**Published:** 2019-12-20

**Authors:** Christopher M. Ranger, Christopher T. Werle, Peter B. Schultz, Karla M. Addesso, Jason B. Oliver, Michael E. Reding

**Affiliations:** 1 USDA-Agricultural Research Service, Horticultural Insects Research Lab, 1680 Madison Ave., Wooster, OH 44691, USA; mike.reding@usda.gov; 2 Department of Entomology, The Ohio State University, Ohio Agricultural Research and Development Center, 1680 Madison Ave., Wooster, OH 44691, USA; 3 USDA-Agricultural Research Service, Thad Cochran Southern Horticultural Lab, 810 Hwy 26 W, Poplarville, MS 39470, USA; chris.werle@usda.gov; 4 Hampton Roads Agricultural Research and Extension Center, Virginia Polytechnic Institute and State University, Virginia Beach, VA 23455, USA; schultzp@vt.edu; 5 Otis L. Floyd Nursery Research Center, Tennessee State University, College of Agriculture, McMinnville, TN 37110, USA; kaddesso@blomand.net (K.M.A.); jasoliver@blomand.net (J.B.O.)

**Keywords:** *Anisandrus maiche*, *Xylosandrus germanus*, Scolytinae, long-lasting insecticide netting, deltamethrin

## Abstract

Ambrosia beetles (Coleoptera: Curculionidae: Scolytinae) are destructive wood-boring insects of horticultural trees. We evaluated long-lasting insecticide netting for protecting stems against ambrosia beetles. Container-grown eastern redbud, *Cercis canadensis*, trees were flood-stressed to induce ambrosia beetle attacks, and deltamethrin-treated netting was wrapped from the base of the stem vertically to the branch junction. Trees were deployed under field conditions in Ohio, Virginia, Tennessee, and Mississippi with the following treatments: (1) flooded tree; (2) flooded tree with untreated netting; (3) flooded tree with treated ‘standard mesh’ netting of 24 holes/cm^2^; (4) flooded tree with treated ‘fine mesh’ netting of 28 holes/cm^2^; and/or (5) non-flooded tree. Treated netting reduced attacks compared to untreated netting and/or unprotected trees in Mississippi in 2017, Ohio and Tennessee in 2018, and Virginia in 2017–2018. Inconsistent effects occurred in Mississippi in 2018. Fewer *Anisandrus maiche*, *Xylosandrus germanus*, and *Xyleborinus saxesenii* were dissected from trees deployed in Ohio protected with treated netting compared to untreated netting; trees deployed in other locations were not dissected. These results indicate long-lasting insecticide netting can provide some protection of trees from ambrosia beetle attacks.

## 1. Introduction

Bark and ambrosia beetles (Curculionidae: Scolytinae) are among the most destructive wood-boring insects of trees growing in a variety of habitats [1,2]. Ambrosia beetles are polyphyletic and therefore not taxonomically distinct from bark beetles, but about 3400 species within the Scolytinae and 1400 species within the Platypodinae are known as ambrosia beetles due to their mutualism with nutritional fungal symbionts [2,3]. Specifically, ambrosia beetles within the tribe Xyleborini represent half of the 60 non-native scolytines in the United States [4], including some key species that attack horticultural trees in nurseries, orchards, and groves [2,5,6].

Adult female Xyleborini tunnel into the sapwood and heartwood of trees to create galleries for cultivating their nutritional fungal symbiont and rearing offspring. The fungal symbiont is introduced into the host tissues during tunnel excavation, and a variety of secondary microorganisms can also be passively introduced [7,8,9]. Discolored sapwood and heartwood tissue often surround the galleries, which may be attributed to the symbiotic fungi, secondary pathogens, host hypersensitive responses, and/or infusion of oxygen into the tissues [5,7,10]. Blockage of the upward movement of water within the stem is likely responsible for branch dieback and tree death following ambrosia beetle attacks [7]. Other indications of an infestation can include toothpick-like extrusions of chewed wood projecting from the stems and sap stains on the bark [5]. Attacks do not always result in plant death, but growth and aesthetic value can be negatively impacted.

Ambrosia beetles, particularly the *Xylosandrus* spp., attack a broad range of trees, but thin-barked deciduous trees are most commonly attacked. Despite an extensive host range, trees emitting ethanol in response to abiotic and biotic stressors are preferentially attacked by a variety of xyleborine ambrosia beetles [5]. A variety of stressors can induce the production and emission of ethanol, including flood stress [11,12], freeze stress [13], and pathogen infection [14], thereby triggering attacks by ambrosia beetles. Maintaining tree health is a fundamental strategy to managing ambrosia beetles in nurseries, orchards, and groves. Yet, stem applications of preventive insecticides are commonly applied because it is often difficult to identify physiologically-stressed trees emitting ethanol before attacks are initiated.

Pyrethroid-based insecticides, including permethrin, cypermethrin, and bifenthrin, are preventively applied to trees for controlling ambrosia beetles and exhibit better efficacy compared to other active ingredients [15,16,17]. However, peak flight of ambrosia beetles occurs during spring months and the timing of preventive insecticide applications coincides with the flowering of many deciduous trees, thereby increasing the potential for non-target impacts on pollinators [16]. Thorough coverage of the stem during insecticide applications is desirable since the majority of exotic xyleborine spp. attacking horticultural trees preferentially attack stems over branches. Considering this aspect of ambrosia beetle host preference behavior, long-lasting insecticide netting was identified as a potential alternative to liquid spray applications of pyrethroid-based insecticides. The netting consists of polyester or polyethylene fabric in which insecticides have been incorporated or coated onto the surface [18]. Pyrethroid-based insecticides are generally used as active ingredients and can be released from the netting over durations of months to years [18,19].

Insecticide-treated netting was initially designed for controlling mosquitoes [18], but has since been used for managing agricultural and forest pests [19,20,21,22,23,24]. Both the Forest Stewardship Council (FSC) and World Health Organization (WHO) have approved the use of treated netting for insect pest management [24,25]. Specifically, Franjević et al. [24] described cypermethrin-treated netting as highly effective for preventing attacks by *Xylosandrus germanus* Blandford and other ambrosia beetles on fresh cut logs in Croatia, further supported in preliminary assessments by Galko et al. [25] with *X. germanus* in Slovakia. Cypermethrin-treated netting was also highly effective at protecting logs of Norway spruce (*Picea abies* L.) from attack by bark beetles in Poland [21].

In the current study, we sought to assess the efficacy of insecticide-treated netting for protecting trees from attack by ambrosia beetles. The overall objective was to determine if deltamethrin-treated netting wrapped around the stems of living trees provided a sufficient barrier for reducing attacks by ambrosia beetles.

## 2. Materials and Methods

Field experiments were conducted in Mississippi in 2017 and 2018, Ohio in 2018, Tennessee in 2018, and Virginia in 2017 and 2018 to evaluate the efficacy of insecticide-treated netting for reducing attacks on deciduous trees. Field sites within or adjacent to woodlots in Mississippi, Ohio, Tennessee, and Virginia were chosen for containing source populations of ambrosia beetles based on previous studies. Container-grown eastern redbud, *Cercis canadensis* L., were locally-sourced for each location and used in all experiments; flood-stress was also used to predispose trees to attack as part of efficacy studies [26]. As noted in the succeeding text, the duration that flood-stressed *C. canadensis* were deployed under field conditions varied among states depending on ambrosia beetle flight activity to ensure adequate attacks occurred on the untreated flooded control trees. Factory-treated black polyethylene netting (deltamethrin, 0.4% active ingredient, 3.85 mg active ingredient/g of netting; Vestergaard Frandsen Inc., Lausanne, Switzerland) was tested during the field experiments. Specific methods used at each study site are described herein.

### 2.1. Mississippi Study Site

**Figure 1:**
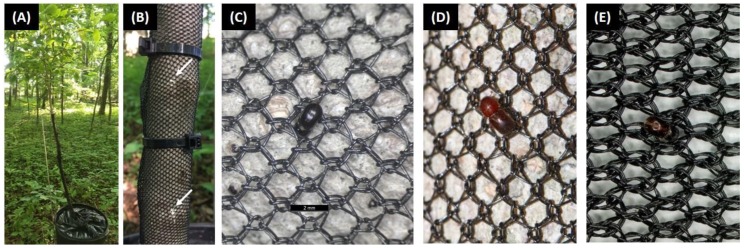
(**A**) *Cercis canadensis* tree deployed in a woodlot in Ohio subjected to flood-stress using a pot-in-pot technique, and with a layer of deltamethrin-treated ‘standard mesh’ covering the main stem. (**B**) Sawdust associated with ambrosia beetle tunneling activity as indicated by the white arrows. (**C**) *Xylosandrus germanus* (scale bar = 2 mm) and (**D**) *Xylosandrus crassiusculus* on ‘standard mesh’ netting with an approximate opening of 1.3 mm × 1.6 mm (l × w). (**E**) *X. germanus* on ‘fine mesh’ netting with an approximate opening of 0.8 mm × 1.5 mm (l × w). Notably, *X. germanus* and *X. crassiusculus* are about 1 mm and 1.2 mm wide, respectively [5].

Container-grown root-grafted *Cercis canadensis* L. were used in Mississippi in 2017 and 2018. Tree root systems were approximately 2 years old when they were transferred to 14.6 L containers with composted pine bark:sand (8:1 v:v; Blow Molded Nursery Container; Nursery Supplies, Chambersburg, PA, USA) and maintained for another 1–2 years of growth. Trees were about 1.5 m in height at the time of experiments. Flood stress conditions were imposed on the *C. canadensis* trees using a pot-in-pot system described by Ranger et al. [11]. In short, a 34.7 L pot was first lined with a plastic waste bag of 3 mil (0.076 mm) thickness. The 14.6 L pot containing a single tree was then placed within the plastic lined pot. Flood stress was imposed by irrigating the media within the internal pot until there was standing water around the base of the tree. Excess plastic liner was tucked in between the two pots to prevent beetles from landing in the standing water (Figure 1A). Standing water was maintained around the base of the flood-stressed trees throughout the duration of the experiments, but non-flooded trees were watered as needed according to standard practices.

In Mississippi, the protective layer of netting was secured to the trees by first pulling the fabric tightly against the stem extending from the base vertically to the base of the lowest branches (about 1 m from base of the stem). The free vertical edges of the netting were then stapled together to secure the netting around the stem, resulting in a gap between the stem and netting. Plastic cable ties also were used to tighten the fabric against the bottom and top regions of the stem to further secure the netting. A ‘standard mesh’ Vestergaard^®^ netting of 24 square holes per cm^2^ was tested in 2017 and 2018 in Mississippi, along with a ‘fine mesh’ Vestergaard^®^ netting of 28 square holes per cm^2^ in 2018.

The following treatments were tested in Mississippi in 2017: (1) flooded tree; (2) flooded tree with untreated ‘standard mesh’ netting; (3) flooded tree with treated ‘standard mesh’ netting; and (4) non-flooded tree (*n* = 6 trees per treatment). Flooding, protective netting, and deployment under field conditions in Mississippi occurred for about 28 days from 7–9 March 2017 to 4–6 April 2017. Flood stress was maintained throughout the duration of the experiment. Trees were arranged in six randomized complete blocks in 2017; two blocks along the edge of a deciduous woodlot in Stone Co., MS, USA (30°47′59.92″ N; 89°15′21.64″ W) and four blocks at a similar site in Pearl River Co., MS, USA (30°65′96.84″ N; 89°63′50.69″ W). Trees within each block were 3 m apart and replicated blocks were separated by 6 m. Flood stress was maintained throughout the duration of the experiment, and the netting was confirmed to be securely in place around the stems. At the end of the experiment, all stems were cut at the base with the netting still in place and transferred to a walk-in refrigerator held at 5 °C, which is cold enough to prevent adults from leaving their galleries and initiating new attacks. Stems were individually removed from the refrigerator and ambrosia beetle attacks on the main stem underneath the netting were recorded, but specimens were not excavated or reared from the infested stems. Attacks were also recorded on the main stem of flooded and non-flooded trees.

The following treatments were tested in Mississippi in 2018: (1) flooded tree; (2) flooded tree with untreated ‘standard mesh’ netting; (3) flooded tree with treated ‘standard mesh’ netting; (4) flooded tree with treated ‘fine mesh’ netting; and (5) non-flooded tree (*n* = 6 trees per treatment). Flooding, protective netting, and deployment under field conditions in Mississippi occurred for about 42 days from 28–29 March 2018 to 9–11 May 2018. Trees were arranged within six randomized complete blocks in 2018 at the same locations. Replicated blocks were arranged linearly with 3 m between adjacent trees within each block, and 6 m between adjacent blocks. Flood stress was maintained throughout the duration of the experiment, and the netting was confirmed to be securely in place around the stems. At the end of the field experiment, all stems were processed as previously described.

### 2.2. Ohio Study Site

*Cercis canadensis* trees were approximately 5 years old, 2.1 m tall, and growing in 23.2 L containers (Haviland Plastics HHP Large Blow Molded Nursery Container; HC7; Haviland, OH, USA) with a mixture of aged pine bark, peat, and coarse sand (60:30:10 v:v:v). Flood stress conditions were imposed on the *C. canadensis* trees using a pot-in-pot system as previously described. In short, a 40.4 L pot (Haviland Plastics HHP; HC10) was first lined with a plastic waste bag of 3 mil (0.076 mm) thickness. A 23.2 L pot (Haviland Plastics HHP; HC7) containing a single tree was then placed within the plastic lined pot. Standing water over the soil line was maintained throughout the duration of experiments.

On the same day trees were deployed under field conditions and flooding was initiated, a layer of black polyethylene netting that was either untreated or treated was tightly wrapped around the stem (Figure 1A). The protective layer of netting extended from the base of the stem vertically to the beginning of the branches (about 90 cm) with some overlap of the netting to prevent exposed stem tissue. Plastic cable ties were then tightened around the stem every 10 cm in vertical increments up from the base to prevent the netting from unwrapping and exposing stem tissue. A ‘standard mesh’ netting was tested in Ohio.

The following treatments were tested in Ohio: (1) flooded tree; (2) flooded tree with untreated ‘standard mesh’ netting; (3) flooded tree with treated ‘standard mesh’ netting; and (4) non-flooded tree (*n* = 6 trees per treatment). Flooding, protective netting, and deployment under field conditions in Ohio occurred for 13 days from 6 June 2018 to 19 June 2018. Trees were arranged in six randomized complete blocks within a mixed deciduous woodlot (40°47′3.13″ N; 81°50′6.21″ W).

Trees within each block were 3 m apart and replicated blocks were separated by 6 m. Flood stress was maintained throughout the duration of the experiment, and the netting was confirmed to be securely in place around the stems. At the end of the field experiment, all stems were stored and attacks recorded as previously described. Stems were also dissected under laboratory conditions and adult ambrosia beetle specimens recovered from host tissues were identified to species and quantified.

### 2.3. Virginia Study Site

Container-grown *C. canadensis* were approximately 6 years old, 2 m tall, and growing in 26.5 L containers with composted pine bark:sand (8:1 v:v; Blow Molded Nursery Container; Nursery Supplies, Chambersburg, PA, USA). Flood stress conditions were imposed using a pot-in-pot system as previously described including 25.6 L and 56.8 L inner and outer pots, respectively. Standing water over the soil line was maintained throughout the duration of experiments.

The protective layer of netting was secured to the trees used in Virginia by first pulling the fabric tightly against the stem extending from the base vertically to the base of the lowest branches. The free vertical edges of the netting were stapled together as previously described for the Mississippi field site. Plastic cable ties were also used to tighten the fabric against the bottom and top regions of the stem to further secure the netting. A ‘standard mesh’ netting was tested in 2017 and 2018 in Virginia, plus a ‘fine mesh’ netting in 2018.

The following treatments were tested in Virginia in 2017: (1) flooded tree; (2) flooded tree with untreated ‘standard mesh’ netting; (3) flooded tree with treated ‘standard mesh’ netting; and (4) non-flooded tree (*n* = 6 trees per treatment). Flooding, protective netting, and deployment under field conditions in Virginia occurred for 28 days from 3 April 2017 to 1 May 2017. Trees were arranged in six randomized complete blocks in 2017 along the edge of a deciduous woodlot at a retail garden center (36°45′32″ N; 76°12′13″ W). Trees within each block were 3 m apart and replicated blocks were separated by 6 m. Flood stress was maintained throughout the duration of the experiment, and the netting was confirmed to be securely in place around the stems. At the end of the field experiment, all stems were stored and attacks recorded as previously described.

The following treatments were tested in Virginia in 2018: (1) flooded tree; (2) flooded tree with untreated ‘standard mesh’ netting; (3) flooded tree with treated ‘standard mesh’ netting; and (4) flooded tree with treated ‘fine mesh’ netting (*n* = 6 trees per treatment). Flooding, protective netting, and deployment under field conditions in Virginia occurred for 22 days from 17 May 2018 to 7 June 2018. Trees were arranged within five randomized complete blocks in 2018 adjacent to a deciduous woodlot (36°53′50″ N; 75°59′37″ W). Replicated blocks were arranged linearly with 3 m between adjacent trees within each block, and 6 m between adjacent blocks. Flood stress was maintained throughout the duration of the experiment, and the netting was confirmed to be securely in place around the stems. At the end of the field experiment, all stems were stored and attacks recorded as previously described.

### 2.4. Tennessee Study Site

Container-grown *C. canadensis* were approximately 3 years old, 1.5 m tall, and growing in 11.3 L black plastic nursery containers (Hummert International, St. Louis, MO, USA) with Pro-Gro Mix (Barky Beaver, Moss, TN, USA; 78% pine bark, 12% peat moss, 10% sand, and 4.8 kg lime/m^3^ with a manufacturer reported bulk density range of 240.3 to 256.3 kg/m^3^) amended with fertilizer (18N-6P-12K Osmocote fertilizer with micronutrients, ICL Fertilizers Company, Dublin, OH, USA) and maintained with overhead irrigation until use in field tests. Flood stress conditions were imposed by submersing the containers in 18.9 L buckets. Standing water over the soil line was maintained throughout the duration of experiments.

The protective layer of netting was secured to the trees used in Tennessee by pulling the fabric tightly against the stem extending from the base vertically to the base of the lowest branches (about 1 m from base of stem). The edges of the netting were secured with staples. Plastic cable ties were used to secure the fabric against the bottom and top of the stem. A ‘standard mesh’ netting and a ‘fine mesh’ netting were evaluated along with an untreated ‘standard mesh’.

The following treatments were tested in Tennessee in 2018: (1) flooded tree; (2) flooded tree with untreated ‘standard mesh’ netting; (3) flooded tree with treated ‘standard mesh’ netting; (4) flooded tree with ‘fine mesh’ netting; and (5) non-flooded tree (*n* = 6 trees per treatment). Flooding, protective netting, and deployment under field conditions in Tennessee occurred for 54 days from 5 May 2018 to 27 June 2018. Trees were arranged within six randomized complete blocks 5 m from a forest edge (35°42′29.21″ N; 85°44′39.41″ W). Replicated blocks were arranged parallel to the forest with 5 m between adjacent trees within each block, and 10 m between adjacent blocks. Flood stress was maintained throughout the duration of the experiment, and the netting was confirmed to be securely in place around the stems. At the end of the field experiment, all stems were stored and attacks recorded as previously described.

### 2.5. Statistics

Count data of ambrosia beetle attacks on flooded trees associated with experiments conducted in Mississippi, Ohio, Tennessee, and Virginia were separately compared using a one-way ANOVA and Fisher’s least significant difference test (α = 0.05; SAS Institute Inc., Cary, NC, USA). Data were square root transformed for analysis to normalize the data, but untransformed means are presented in the original scale of measurement for presentation [27]. Similarly, ambrosia beetle specimens recovered from trees deployed in Ohio were square root transformed and analyzed using one-way ANOVA and Fisher’s least significant difference test (α = 0.05) with untransformed means being presented.

## 3. Results

### 3.1. Mississippi Study Site

**Figure 2:**
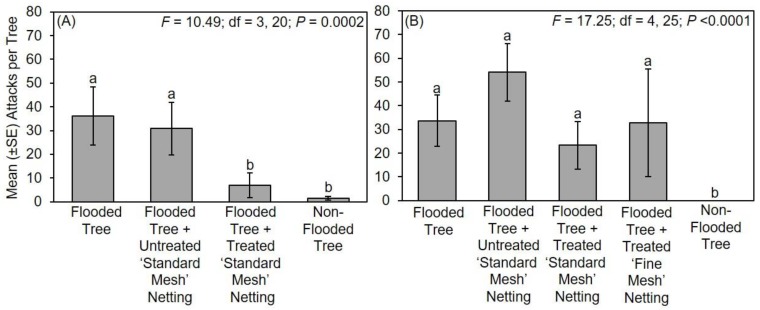
Impact of long-lasting insecticide netting for protecting flood-stressed and non-flooded *C. canadensis* trees deployed in Mississippi in (**A**) 2017 and (**B**) 2018. A ‘standard mesh’ netting was tested in 2017, and ‘standard mesh’ and ‘fine mesh’ netting were tested in 2018. Means ± standard error (SE) with different letters are significantly different (one-way ANOVA; Fisher’s least significant difference (LSD) test).

During the 2017 experiment, significantly fewer attacks occurred on flooded trees protected with treated ‘standard mesh’ netting and non-flooded trees compared to flooded trees without protective netting and flooded trees with untreated ‘standard mesh’ netting (Figure 2A). In particular, non-flooded trees without netting sustained a mean ± standard error (SE) of 1.5 ± 0.8 attacks per tree, flooded trees deployed with treated ‘standard mesh’ netting sustained 7.0 ± 5.2 attacks per tree, flooded trees with untreated ‘standard mesh’ netting sustained 30.8 ± 11.1 attacks per tree, and flooded trees deployed without protective netting sustained a mean ± SE of 36.2 ± 12.3 attacks per tree.

During the 2018 experiment, significantly fewer attacks occurred for non-flooded trees compared to all the remaining treatments, namely, flooded trees without netting, flooded trees with untreated ‘standard mesh’ netting, and flooded trees with treated ‘standard mesh’ or ‘fine mesh’ netting (Figure 2B). There was no difference in the number of attacks to flooded trees with treated or untreated mesh. In particular, no attacks occurred on non-flooded trees, flooded trees with treated ‘fine mesh’ netting sustained a mean ± SE of 32.8 ± 22.6 attacks per tree, flooded trees with treated ‘standard mesh’ netting sustained 23.3 ± 10.0 attacks per tree, flooded trees with untreated ‘standard mesh’ netting sustained 54.2 ± 12.1 attacks per tree, and unprotected flooded trees sustained 33.7 ± 10.8 attacks per tree.

### 3.2. Ohio Study Site

**Figure 3:**
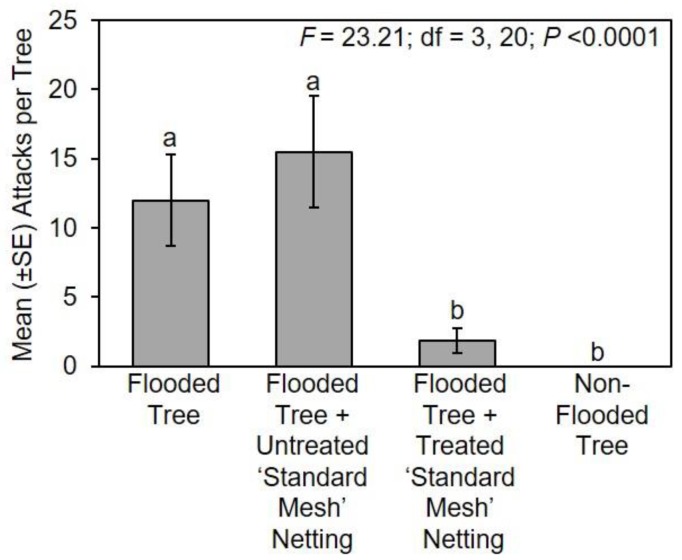
Impact of long-lasting insecticide netting for protecting stems of flood-stressed and non-flooded *C. canadensis* trees deployed in Ohio in 2018. A ‘standard mesh’ netting coated with deltamethrin was tested. Means with different letters are significantly different (one-way ANOVA; Fisher’s LSD test).

During the 2018 experiment, significantly fewer attacks occurred on flooded *C. canadensis* trees protected with treated ‘standard mesh’ netting and non-flooded trees compared to flooded trees and flooded trees with untreated netting (Figure 1 and Figure 3). In particular, no attacks occurred on non-flooded trees, and flooded trees with treated ‘standard mesh’ netting sustained a mean ± SE of 1.8 ± 0.9 attacks per tree. By contrast, stems of flooded trees with untreated ‘standard mesh’ netting sustained 15.5 ± 4.0 attacks per tree, and flooded trees deployed without protective netting sustained a mean ± SE of 12.0 ± 3.3 attacks per tree.

A mean ± SE of 66.3 ± 14.7 percent of attacks on flooded trees without netting were occupied by scolytine foundresses (i.e., 57 occupied tunnels per 72 total attacks), 79.0 ± 7.5 percent of attacks on flooded trees with untreated netting were occupied by foundresses (i.e., 81 occupied tunnels per 93 total attacks), and 93.3 ± 6.7 percent of attacks on flooded trees with treated netting were occupied by foundresses (i.e., 10 occupied tunnels out of 11 total attacks). Overall, a total of 55 adult ambrosia beetles was recovered from stems of flooded trees, 81 specimens from stems with untreated netting, 10 specimens from stems of trees protected by treated netting, and 0 specimens from stems of non-flooded trees (Table 1). A total of 78 *Anisandrus maiche* Stark, 40 *X. germanus*, 26 *Xyleborinus saxesenii* Ratzeburg, and 2 *Ambrosiodmus rubricollis* (Eichhoff) were recovered from the infested *C. canadensis* trees.

Significantly fewer Scolytinae specimens pooled across species were recovered from stems of flooded trees protected with treated netting and non-flooded trees compared to stems of flooded trees without protective netting and flooded trees with untreated netting (Table 1). Furthermore, significantly fewer *A. maiche*, *X. germanus*, and *X. saxesenii* were recovered from stems of flooded trees protected with treated netting compared to flooded trees with untreated netting (Table 1).

### 3.3. Tennessee Study Site

**Figure 4:**
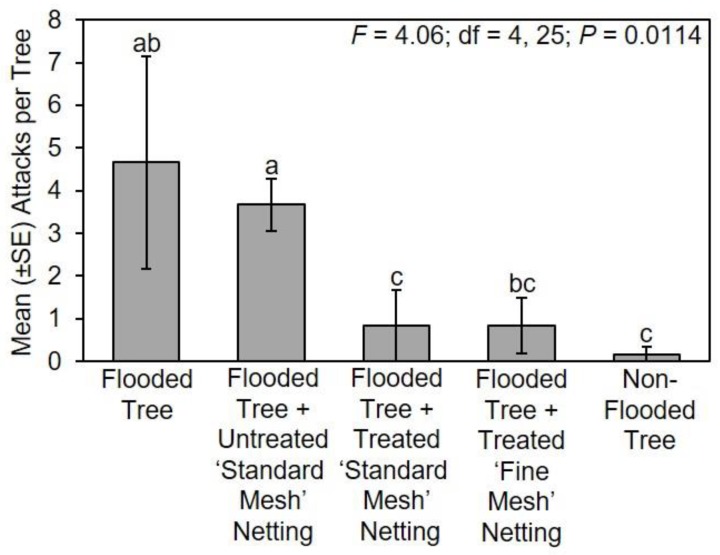
Impact of long-lasting insecticide netting for protecting flood-stressed and non-flooded *C. canadensis* trees deployed in Tennessee in 2018. A ‘standard mesh’ and ‘fine mesh’ netting were both tested in 2018. Means with different letters are significantly different (one-way ANOVA; Fisher’s LSD test).

During the 2018 experiment, significantly fewer attacks occurred on *C. canadensis* that were flooded and wrapped with treated ‘standard mesh’ netting or ‘fine mesh’ netting and non-flooded trees compared to flooded trees with untreated ‘standard mesh’ netting (Figure 4). There was no difference in the number of attacks on unprotected flooded trees compared to flooded trees with untreated ‘standard mesh’ netting. There was also no difference in the number of attacks to flooded trees without netting compared to flooded trees with treated ‘fine mesh’ netting. Non-flooded trees sustained a mean ± SE of 0.17 ± 0.17 attacks per tree, flooded trees with treated ‘fine mesh’ netting sustained 0.83 ± 0.65 attacks per tree, flooded trees with treated ‘standard mesh’ sustained 0.83 ± 0.83 attacks per tree, flooded trees with untreated ‘standard mesh’ netting sustained 3.67 ± 0.61 attacks per tree, and unprotected flooded trees sustained 4.67 ± 2.49 attacks per tree (Figure 4).

### 3.4. Virginia Study Site

**Figure 5:**
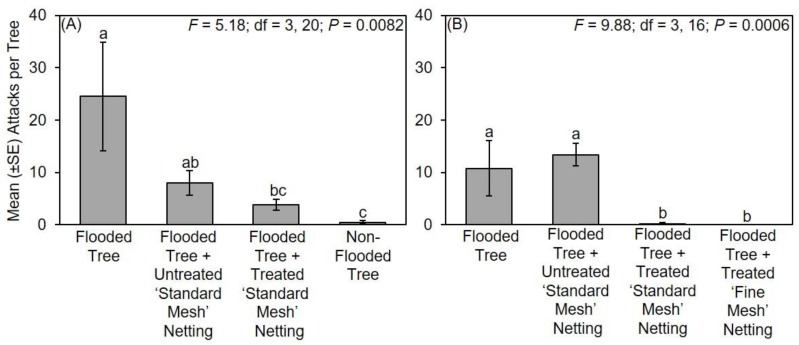
Impact of long-lasting insecticide netting for protecting flood-stressed and non-flooded *C. canadensis* trees deployed in Virginia in (**A**) 2017 and (**B**) 2018. A ‘standard mesh’ netting was tested in 2017, and ‘standard mesh’ and ‘fine mesh’ netting were tested in 2018. Means with different letters are significantly different (one-way ANOVA; Fisher’s LSD test).

During the 2017 experiment, significantly fewer attacks occurred on flooded *C. canadensis* trees with treated ‘standard mesh’ netting compared to unprotected flooded trees (Figure 5A). In particular, stems of non-flooded trees sustained a mean ± SE of 0.5 ± 0.3 attacks per tree, flooded trees protected by treated ‘standard mesh’ netting sustained 3.8 ± 1.1 attacks per tree, flooded trees with untreated ‘standard mesh’ netting sustained 8.0 ± 2.3 attacks per tree, and flooded trees sustained a mean ± SE of 24.5 ± 10.4 attacks per tree.

During the 2018 experiment, significantly fewer attacks occurred on flooded trees protected with treated ‘standard mesh’ and ‘fine mesh’ netting compared to flooded trees with and without untreated netting (Figure 5B). There was no difference in attacks to trees protected with treated ‘standard mesh’ and treated ‘fine mesh’ netting. In particular, stems of flooded trees with treated ‘fine mesh’ netting sustained no attacks, and stems of flooded trees deployed with treated ‘standard mesh’ netting sustained a mean ± SE of 0.2 ± 0.2 attacks per tree. By contrast, stems of flooded trees deployed with untreated ‘standard mesh’ netting sustained 13.4 ± 2.2 attacks per tree, and stems of unprotected flooded trees sustained 10.8 ± 5.2 attacks per tree (Figure 5B).

## 4. Discussion

In multi-state field trials, deltamethrin-treated netting showed variable efficacy at protecting stems of flood-stressed *C. canadensis* from attack by ambrosia beetles. Specifically, a reduction in attacks to stems protected by treated netting was observed in Mississippi in 2017 trials, and Ohio, Tennessee, and Virginia in 2018, but not Mississippi in 2018 or Virginia in 2017. The basis for inconsistent efficacy across years and/or locations for protecting stems from ambrosia beetles remains unclear. Studies by Franjević et al. [24] found cypermethrin-treated netting provided nearly complete protection of oak logs from *X. germanus* and other ambrosia beetles. Similarly, no bark beetle galleries were detected in any Norway spruce logs protected by cypermethrin-treated netting [21]. Previous studies demonstrated *X. germanus* and other ambrosia beetles readily attack trees emitting ethanol despite the presence of insecticide residues on the bark surface [15,16,26]. While showing promise, the insecticide-treated netting tactic will therefore need to be further optimized for protecting stems of valuable trees from ambrosia beetles. Additional studies are warranted to compare deltamethrin-treated netting with other active ingredients, such as cypermethrin-treated netting, which also are available [19,21,22].

As the deltamethrin provided some degree of a chemical barrier, there was speculation during our current study that the ‘standard mesh’ netting did not provide a satisfactory physical barrier against ambrosia beetles, unlike the Japanese beetle *Popillia japonica* Newman [19] and brown marmorated stink bug *Halymorpha halys* [22,23]. Two of the most problematic ambrosia beetles in ornamental nurseries and tree fruit orchards, *X. germanus* and *X. crassiusculus*, are about 1 mm and 1.2 mm wide, respectively [5]. Both *X. germanus* and *X. crassiusculus* were able to pass through the opening in the ‘standard’ and ‘fine’ mesh without chewing (Figure 1C,D), but more contact occurred between the cuticle and ‘fine’ mesh (Figure 1E). However, subsequent experiments conducted in Virginia and Mississippi in 2018 which did not find a ‘fine mesh’ netting with an opening of 0.8 mm × 1.5 mm (l × w) provided improved protection compared to a ‘standard mesh’ size opening of 1.3 mm × 1.6 mm (l × w). It is likely these beetles would have had some level of contact with the netting during boring activity to enter the tree, but it is possible the exposure duration was insufficient in some instances to induce mortality. It is also unclear if the durations and/or behaviors involved with landing, movement, and boring among the different ambrosia beetle species may expose them to less dislodgeable residues from the treated netting. Ambrosia beetles may also chew through insecticide-treated netting to attack stems emitting ethanol; for instance, *Cnestus mutilatus* (Blandford) bored into plastic containers storing gasoline and a 10% ethanol component [28].

Maintaining tree health to minimize the production and emission of ethanol, and risk of ambrosia beetle attack, is further evident by the few to no attacks that occurred on non-flooded *C. canadensis* trees deployed as part of our current study. Additionally, results from our current study support previous research [6,11,12,26,29,30] that flood-stress of container-grown trees is a useful tactic for predisposing trees to attack by ambrosia beetles. For example, flooding of *C. candensis* and *Cornus florida* L. facilitated insecticide and fungicide efficacy trials targeting ambrosia beetles and their nutritional fungal symbiont [26]. Similarly, flooding of *C. canadensis* and *Liriodendron tulipifera* L. was used during field trials assessing the interaction between preventive fungicide treatment and root infection by *Phytophthora cinnamomi* on ambrosia beetle attacks [30]. Flooding of *Malus domestica* Borkh was used for evaluating insecticides against *X. germanus* and other ambrosia beetles [6].

An important consideration when using flood-stress to induce ethanol production and ambrosia beetle attacks is using tree species with known intolerance of flooding over moderately tolerant or tolerant species [11,12]. Additionally, using a pot-in-pot system and ensuring that standing water is maintained over the root system throughout the duration of the experiment is critical to minimizing oxygen uptake by the roots and inducing anaerobic respiration and ethanol production [11,12,31,32,33]. Failure to maintain standing water throughout the duration of the experiment can disrupt the transition from aerobic to anaerobic respiration and the subsequent production of ethanol. Finally, since ethanol is produced within the roots of flood-stressed plants and then transported to the stem and leaf tissues [31,32,33], it is also important to use trees with a vigorous, well-established root system to maximize tree attractiveness to ambrosia beetles. Using these aforementioned techniques, ethanol was detected within flood-intolerant trees tissues at 3, 7, and 14 days after initiating flooding, and appreciable numbers of attacks on flood-intolerant trees occurred by 14 to 21 days after initiating flooding [11,12].

Dissection of tree stems deployed in Ohio demonstrated the exotic species *A. maiche* and *X. germanus* were the two most abundant species responsible for initiating attacks. *Xylosandrus germanus* is a dominant exotic species in Ohio and upper Midwestern US [5], but the distribution of *A. maiche* has increased since first being reported in North America in 2009 [34,35]. The incidence of *A. maiche* in experimentally-stressed trees deployed in Ohio has also increased [12,13,36]. Notably, caution must be taken to accurately distinguish between *A. maiche* and *X. germanus* since their morphology is very similar, except the procoxae of *A. maiche* are contiguous [34,35].

Inconsistent with experiments conducted in Ohio, stems of flood-stressed trees deployed in Mississippi, Tennessee, and Virginia were not dissected as part of our current study. Thus, it is unclear what species of ambrosia beetles were responsible for tree attacks within these regions. Still, previous dissections of experimentally-stressed trees found that *Xylosandrus crassiusculus* (Motschulsky) and *X. germanus* were the dominant species recovered in Virginia [13,26,36]; *Cnestus mutilatus* and *X. crassiusculus* were the dominant species recovered in Tennessee [37]; and *Hypothenemus dissimilis* (Zimmermann), *Xylosandrus compactus* (Eichhoff), and *X. crassiusculus* were the dominant species in Mississippi [36]. Differences in the seasonal activity and composition of Xyleborine ambrosia beetles could account for variability in efficacy of the insecticide treated netting observed between years and locations. As such, future studies involving ambrosia beetles and stressed trees should include dissections of infested host tissues to test for interspecific differences.

Based on our current study, insecticide-treated netting could ultimately be useful for long-term protection of tree stems/trunks from attack by ambrosia beetles, including trees growing in ornamental nurseries, tree fruit and nut orchards, avocado groves, and high-value landscape specimen trees. While not tested as part of our current study, additional studies are warranted to determine if a fence-type barrier of insecticide-treated netting baited with an ambrosia beetle attractant (i.e., ethanol) around the perimeter of a nursery or susceptible trees could potentially be useful as an attract-and-kill tactic. Ambrosia beetles disperse from woodlots into adjacent nurseries/orchards in search of vulnerable host trees to attack [38,39], potentially making a perimeter barrier effective. The tendency of *X. germanus* and *X. crassiusculus* to fly relatively low to the ground when dispersing from woodlots into ornamental nurseries [40] might enhance the effectiveness of an insecticide-treated barrier fencing. For instance, cypermethrin- and deltamethrin-treated netting baited with a pheromone for *H. halys* posed a chemical and physical barrier when deployed as a fence adjacent to rows of pear trees, *Pyrus* sp. [22,23].

## 5. Conclusions

Our current study provides an initial basis that insecticide-treated netting could be a useful component of an integrated pest management strategy for managing ambrosia beetle pests of horticultural trees. Evidence was obtained, albeit inconsistent, that deltamethrin-treated netting provided a chemical barrier to ambrosia beetles. Support for previous studies also was obtained that flood-stress is a useful tactic to evaluate insecticide efficacy against ambrosia beetles, along with the importance of maintaining tree health to minimize the risk of attack by ambrosia beetles. Additional studies are warranted in an attempt to improve the tactic, for instance, comparing deltamethrin with other active ingredients, assessing a finer mesh size to pose a physical barrier against ambrosia beetles, and testing mesh colors other than black to reduce the visual silhouette of a tree. However, it is unknown if a mesh size smaller than 0.8 mm × 1.5 mm (l × w) is available or will be manufactured for research purposes to exclude ambrosia beetles that are <1 mm in width. Assessing the utility of insecticide-treated and ethanol-baited netting as a barrier fence around vulnerable trees also is warranted. Modifying the color of the netting could also provide an additional form of optimization since Werle et al. [41] demonstrated black or red traps were more attractive to ambrosia beetles than white traps.

## Figures and Tables

**Table 1:** Adult ambrosia beetle specimens excavated from *C. canadensis* trees deployed in Ohio in 2018 with and without a protective layer over the stem of deltamethrin-treated ‘standard mesh’ netting.

	Mean (±SE) Specimens Recovered per Tree									
Treatment	*Anisandrus maiche*		*Xylosandrus germanus*		*Xyleborinus saxesenii*		*Ambrosiodmus rubricollis*		Pooled Scolytinae	
Flooded tree	4.8	±2.4 ab	3.5	±0.9 a	0.7	±0.3 ab	0.2	±0.2 a	9.2	±2.8 a
Flooded tree + untreated netting	6.7	±2.5 a	3.0	±0.5 a	3.7	±1.9 a	0.2	±0.2 a	13.5	±4.2 a
Flooded tree + treated netting	1.5	±0.7 bc	0.2	±0.2 b	0.0	±0.0 b	0.0	±0.0 a	1.7	±0.8 b
Non-flooded tree	0.0	±0.0 c	0.0	±0.0 b	0.0	±0.0 b	0.0	±0.0 a	0.0	±0.0 b
*F*; *P*	5.23; 0.0079		20.90; <0.0001		3.48; 0.035		0.67; 0.58		12.12; <0.0001	

Means ± standard error (SE) with different letters within a column are significantly different using one-way ANOVA and Fisher’s LSD test (df = 3, 20 for all comparisons; *n* = 6 trees per treatment).
